# Metabolic Engineering of *Escherichia coli* for the Production of Xylonate

**DOI:** 10.1371/journal.pone.0067305

**Published:** 2013-07-05

**Authors:** Yujin Cao, Mo Xian, Huibin Zou, Haibo Zhang

**Affiliations:** Key Laboratory of Bio-based Materials, Qingdao Institute of Bioenergy and Bioprocess Technology, Chinese Academy of Sciences, Qingdao, China; University of Houston, United States of America

## Abstract

Xylonate is a valuable chemical for versatile applications. Although the chemical synthesis route and microbial conversion pathway were established decades ago, no commercial production of xylonate has been obtained so far. In this study, the industrially important microorganism *Escherichia coli* was engineered to produce xylonate from xylose. Through the coexpression of a xylose dehydrogenase (*xdh*) and a xylonolactonase (*xylC*) from *Caulobacter crescentus*, the recombinant strain could convert 1 g/L xylose to 0.84 g/L xylonate and 0.10 g/L xylonolactone after being induced for 12 h. Furthermore, the competitive pathway for xylose catabolism in *E. coli* was blocked by disrupting two genes (*xylA* and *xylB*) encoding xylose isomerase and xylulose kinase. Under fed-batch conditions, the finally engineered strain produced up to 27.3 g/L xylonate and 1.7 g/L xylonolactone from 30 g/L xylose, about 88% of the theoretical yield. These results suggest that the engineered *E. coli* strain has a promising perspective for large-scale production of xylonate.

## Introduction

Xylonate is a five-carbon organic acid. In the past few years, xylonate has gained increasing interest due to its potential as an important platform chemical. Xylonate has extensively versatile applications similar to many other sugar acids such as gluconate, and can be used in food, chemical, and pharmaceutical industries [1]. In particular, xylonate could serve as a precursor for 1,2,4-butanetriol synthesis [2] and a concrete water reducer [3]. Xylonate might be produced from the non-food hemicellulose hydrolysate, which provides an inexpensive alternative to gluconate. In a report from the U.S. Department of Energy, xylonate is among the top 30 value-added chemicals manufactured from biomass [4].

Although chemical oxidation of xylose to produce xylonate could be obtained by using platinum or gold as the catalysts [5], the poor selectivity makes these synthetic routes not economically feasible for industrial purposes. Microbial conversion of xylose to xylonate, which was well characterized in previous studies, has become a research hotspot during recent years. Several bacterial strains, e.g., *Enterobacter cloacae* [6], *Pseudomonas fragi* [7], *Gluconobacter oxydans* [8], were able to produce xylonate in high yields. Compared with the chemical synthetic routes, microbial fermentation offers the potential for benign processing with high specificity and reduced manufacturing cost.

Along with the development of modern molecular biology techniques, the metabolic pathway from xylose to xylonate has been largely elucidated. Xylose is first converted to xylonolactone by xylose dehydrogenase or glucose oxidase, and xylonolactone is subsequently hydrolyzed spontaneously or enzymatically by xylonolactonase to yield xylonate [9]. Up to now, genes encoding xylose dehydrogenases have been identified and cloned from different bacterial and fungal strains [10]–[12]. Heterologous expression of these xylose dehydrogenase led to xylonate production in the corresponding hosts [13], [14]. However, few xylonolactonases have been characterized. Spontaneous hydrolysis of xylonolactone was relatively slow, and its accumulation in the culture broth greatly inhibited the growth of bacteria, thus hampering xylonate production [15].

**Figure 1:**
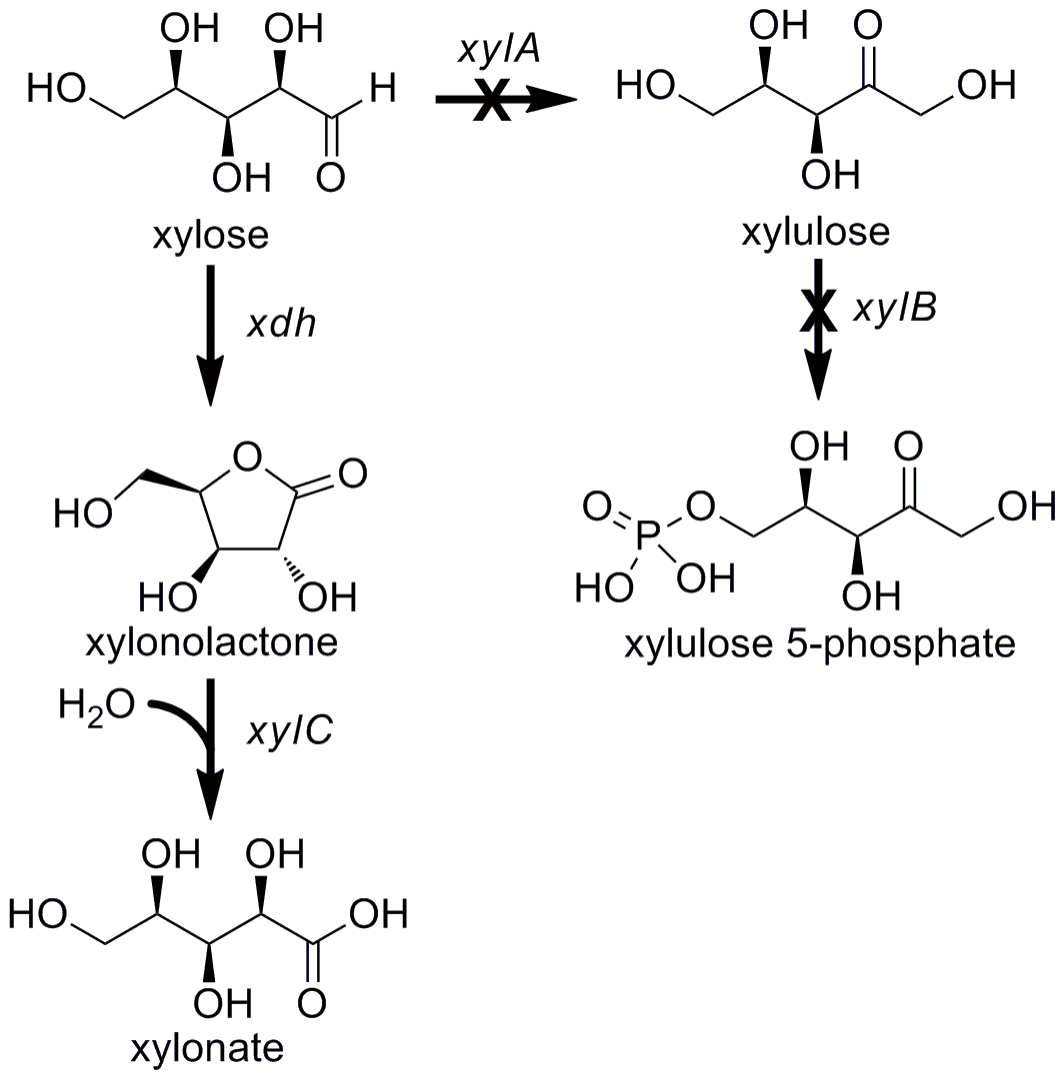
The metabolic pathway from xylose to xylonate in engineered *E. coli*. Enzymes encoded by the genes shown are: *xdh*, xylose dehydrogenase from *C. crescentus*; *xylC*, xylonolactonase from *C. crescentus*; *xylA*, native *E. coli* xylose isomerase; *xylB*, native *E. coli* xylulose kinase.

Recently, the oxidative xylose utilization pathway was discovered in the fresh water bacterium *Caulobacter crescentus*. Enzymes including an NAD^+^ dependent xylose dehydrogenase (*xdh*) and a xylonolactonase (*xylC*) were identified in a xylose-inducible operon [16]. In this study, the two genes were expressed in a xylose catabolic deficient *Escherichia coli* mutant strain (knockout of *xylA* and *xylB*, encoding xylose isomerase and xylulose kinase) to construct a heterologous xylonate-producing system (Figure 1). Xylonate production by the engineered strain was further evaluated under fed-batch culture conditions.

## Materials and Methods

### Bacterial Strains and Plasmids Construction

A list of bacterial strains and recombinant plasmids used in this study is presented in Table 1. The one-step gene inactivation strategy, previously described by Datsenko and Wanner [17], was applied to knock out the chromosomal genes in *E. coli* BL21 star(DE3). Oligonucleotide primers used for gene disruption are listed in Table S1. For the construction of strain BL21ΔxylAB, a linear DNA fragment containing the FRT-flanked kanamycin resistance cassette was amplified with primers xylAB_Del_F and xylAB_Del_R from plasmid pKD4. The obtained disrupting fragments were electrotransformed into *E. coli* competent cells that carried the Red recombinase expression vector pKD46 and integrated into its chromosome. Successfully disrupted colonies were then transformed with plasmid pCP20 and induced at 42°C to eliminate the kanamycin resistance. PCR verifications were performed using primer pairs designed according to the sequences up- and downstream of disrupted regions (xylAB_DelIden_F and xylAB_DelIden_R).

**Table 1:** Strains and plasmids used in this study.

Strains or plasmids	Genotype/Description	Sources
Strains		
*E. coli* BL21 star(DE3)	*F^–^ ompT hsdS_B_* (r_B_^–^ m_B_^–^) *gal dcm rne131* (DE3)	Invitrogen
*E. coli* BL21 star(DE3) ΔxylAB	Knockout of *xylA* and *xylB* encoding xylose isomerase and xylulose kinase	This study
Plasmids		
pACYCduet-1	*Cm^r^ oriP15A lacI^q^ T7p*	Novagen
pA-xdh	pACYCduet-1 harboring *C. crescentus xdh*	This study
pA-xylC	pACYCduet-1 harboring *C. crescentus xylC*	This study
pA-xdhxylC	pACYCduet-1 harboring *C. crescentus xdh* and *xylC*	This study
pKD46	*Ap^r^ oriR101 repA*(Ts) *λ Red* (*γ*, *β* and *exo*)	Coli Genetic Stock Center
pKD4	*FRT-Kan^r^-FRT oriR6K*	Coli Genetic Stock Center
pCP20	*Ap^r^ Cm^r^ repA*(Ts) *FLP*	Coli Genetic Stock Center

The *xdh* (GenBank Accession No.: NACL94329) and *xylC* (GenBank Accession No.: NACL94328) genes from *C. crescentus* were codon optimized, chemically synthesized and cloned into pGH vector by Generay Biotech Co., Ltd. (Shanghai, China). Then *xdh* and *xylC* were PCR amplified and cloned into the restriction sites *Nco*I/*BamH*I and *Nde*I/*Xho*I of vector pACYCduet-1, creating pA-xdh and pA-xylC, respectively. The primers for the amplification of *xdh* and *xylC* are also given in Table S1. The *xdh* gene was further cloned into pA-xylC between *Nco*I and *BamH*I sites leading to plasmid pA-xdhxylC, which was used for the coexpression of the two genes. Successful gene cloning was verified by colony PCR, restriction mapping and direct nucleotide sequencing.

### Reagents, Media and Culture Conditions

Calcium xylonate hydrate for reference standard was purchased from Toronto Research Chemicals Inc. (North York, Canada). Xylonolactone was prepared by autoclaving calcium xylonate in 1 M HCl at 121°C for 20 min [18]. Luria-Bertani (LB) medium (10 g/L tryptone, 5 g/L yeast extract, 10 g/L NaCl) or M9 mineral medium (6 g/L Na_2_HPO_4_, 3 g/L KH_2_PO_4_, 1 g/L NH_4_Cl, 0.5 g/L NaCl, 0.12 g/L MgSO_4_) supplemented with appropriate carbon sources were used for DNA manipulation, protein expression and liquid growth tests. All cultures were grown at 37°C in the presence of 34 µg/ml chloramphenicol and shaken at 180 rpm. 0.5 mM of isopropyl-β-D-thiogalactopyranoside (IPTG) was added at an OD_600_ of 0.6 to induce the expression of recombinant proteins. For xylonate production, xylose was added to the culture to a final concentration of 1 g/L at the same time. Sample was taken at different intervals and the supernatants were used for xylonate and xylonolactone determination.

### Protein Expression and Enzyme Activity Determination

Recombinant *E. coli* strains harboring pA-xdh, pA-xylC or pA-xdhxylC were induced for 4 h to express the recombinant proteins. Cells were collected from 1 ml of bacteria cultures by centrifugation, dissolved in 100 µl sodium dodecyl sulfate (SDS) sample buffer, heated at 100°C for 10 min and then analyzed by SDS-polyacrylamide gel electrophoresis (PAGE) [19]. The harvested bacterial cells were also suspended in phosphate buffer (pH 8.0) and subjected to ultrasonication. The mixture was centrifuged at 12,000 rpm and 4°C for 10 min, and the supernatant obtained was used for enzymatic activity determination. Generally, assays of xylose dehydrogenase were carried out in 1 ml of reaction system containing 50 mM phosphate buffer (pH 8.0), 10 mM xylose, 1 mM NAD^+^ and 10 µl crude protein extracts. The assay mixture containing 50 mM phosphate buffer (pH 8.0), 10 mM xylonolactone, and 10 µl crude protein extracts was used for xylonolactonase enzyme reaction. Activities of the two enzymes were measured by directly monitoring product formation by ion chromatography.

### Fed-batch Fermentation

For large-scale production of xylonate, fed-batch cultures were carried out in a Biostat B plus MO5L fermentor (Sartorius, Germany) containing 3 L of growth medium (20 g/L of tryptone, 10 g/L yeast extract and 5 g/L NaCl) that was sterilized at 121°C for 20 min. Glycerol (10 g/L), K_2_HPO_4_·3H_2_O (5 g/L), MgSO_4_ (0.12 g/L) and trace elements (1 ml per liter, 3.7 g/L (NH_4_)_6_Mo_7_O_24_·4H_2_O, 2.9 g/L ZnSO_4_·7H_2_O, 24.7 g/L H_3_BO_3_, 2.5 g/L CuSO_4_·5H_2_O, 15.8 g/L MnCl_2_·4H_2_O) were autoclaved or filter-sterilized separately and added prior to initiation of the fermentation. 50 ml of inoculum was prepared by incubating the culture in shake flasks containing liquid LB medium overnight at 37°C. The fermentation was first operated in a batch mode and the control settings were: 37°C, stirring speed at 600 rpm, and airflow at 2 L/min. During the fermentation process, the pH was controlled at 7.0 via automated addition of 5 M NaOH. Antifoam 204 was added to prohibit foam development. The agitation was associated with the dissolved oxygen (DO) to maintain a DO concentration of above 20% saturation. After the initial glycerol was nearly exhausted, fed-batch mode was commenced by feeding a solution containing 50% of glycerol at appropriate rates and the residual glycerol was maintained at a very low level. When the cells were grown to an OD_600_ of about 20, IPTG was added to the culture at a concentration of 0.5 mM, xylose was added to a final concentration of 30 g/L, and the culture temperature was switching to 30°C. Samples of fermentation broth were removed at appropriate intervals to determine residual xylose, xylonate and xylonolactone production.

### Determination of Xylose, Xylonolactone and Xylonate by Ion Chromatography

The determination of xylose, xylonolactone and xylonate in the culture broth was performed on an ICS-3000 (Dionex, Sunnyvale, CA, USA) ion chromatography (IC) system. The IC was equipped with an IonPac AS11 anion chromatography column (4.0 mm×250 mm) and an AG-11 guard column (4.0 mm×50 mm). Suppression was achieved with anion suppressor (ASRS 300 4 mm). Peaks were detected using electrochemical detector. A mixture of 250 mM NaOH (2%) and H_2_O (98%) was used for elution at a flow rate of 1 mL/min. For sample analysis, another elution step with 80% of 250 mM NaOH was employed to remove the residual components. Data collection and handling were carried out by Dionex Chromeleon software.

## Results and Discussion

### Inactivation of Native E. Coli Xylose Catabolic Pathways

*E. coli* BL21 star(DE3) was chosen as the host for xylonate production in this work. Unlike *E. coli* K12, strain BL21 and its derivatives lack the requisite xylonate dehydratase activity (potentially encoded by *yjhG* and *yagF*). Therefore, they cannot consume xylonate as the carbon source and would be useful for the accumulation of xylonate in the culture broth. In addition, strain BL21 star(DE3) carries a mutated *rne* gene which encodes a truncated RNase E enzyme and lacks the ability to degrade mRNA, resulting in an increase in mRNA stability. This might be helpful to the Red recombination enzyme activities and thus this strain has been successfully used for gene knockout [20]. *E. coli* could efficiently utilize xylose as a carbon source for growth. The first two genes responsible for xylose catabolism have been recognized as xylose isomerase (encoded by *xylA*) and xylulose kinase (encoded by *xylB*) [21]. In order to block the native xylose catabolic pathway, we disrupted *xylA* and *xylB* in strain BL21 star(DE3) chromosome using the Red recombination method. Successful gene disruptions were confirmed by PCR amplification (Figure S1). Liquid growth tests on M9 mineral medium supplemented with xylose as the sole carbon source further proved that the xylose metabolism-blocked strain BL21ΔxylAB grew much more poorly than its parent strain.

### Heterologous Expression of Xylose Dehydrogenase and Xylonolactonase

**Figure 2:**
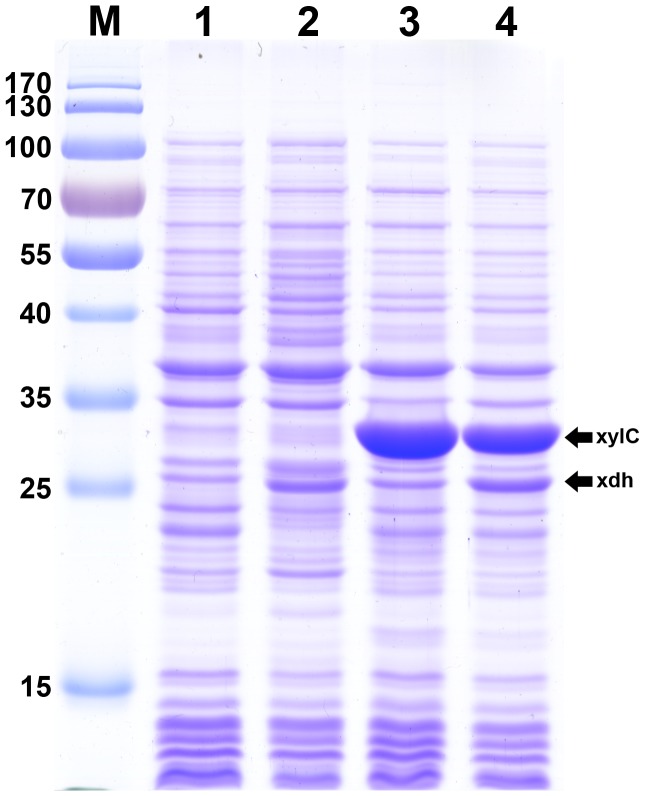
Expression of the recombinant xylose dehydrogenase and xylonolactonase from *C. crescentus*. Lane M, prestained protein ladder; lane 1, BL21ΔxylAB harboring pACYCduet-1; lane 2, BL21ΔxylAB harboring pA-xdh; lane 3, BL21ΔxylAB harboring pA-xylC; lane 4, BL21ΔxylAB harboring pA-xdhxylC.

With the aim to express the xylose dehydrogenase and xylonolactonase enzymes, we cloned the coding region of *xdh* and *xylC* from *C. crescentus* into pACYCduet-1 expression vector under T7 promoter, respectively. The recombinant plasmids pA-xdh, pA-xylC and pA-xdhxylC were confirmed by restriction enzyme digestion and DNA sequencing. To verify the expression levels of the recombinant proteins, strain BL21ΔxylAB was transformed by the expression vectors including different genes and grown in liquid LB medium followed by induction using 0.5 mM IPTG. Figure 2 showed the gel electrophoresis patterns of samples from different recombinant strains analyzed with coomassie brilliant blue staining. We noted distinct bands of the expected size from protein extracts of the recombinant strains compared with the control strain harboring pACYCduet-1. SDS-PAGE analysis of the recombinant strain carrying pA-xdh and pA-xylC revealed the recombinant proteins of different sizes (corresponding to the bands of molecular weight 26.6 kDa and 31.6 kDa) while the lysate of strain BL21ΔxylAB/pA-xdhxylC gave both of the two bands.

### Establishment of a Xylose, Xylonate and Xylonolactone Analysis Method for the Determination of Xylose Dehydrogenase and Xylonolactonase Activities

**Figure 3:**
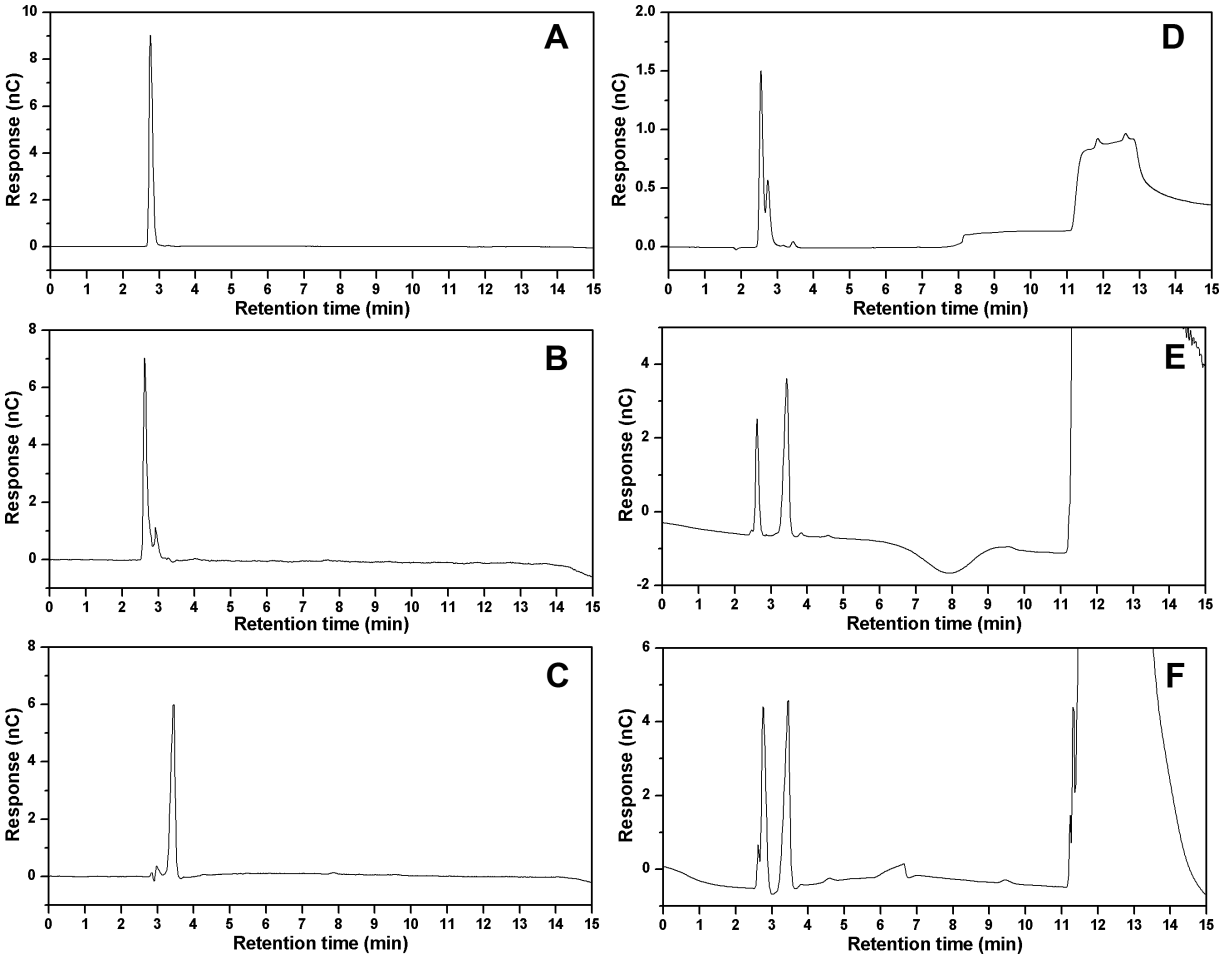
Detection of xylose, xylonate and xylonolactone by ion chromatography. A, 1 ppm xylose, corresponding to the retention time of 2.75 min; B, 200 ppm xylonolactone, corresponding to the retention time of 2.61 min; C, 100 ppm xylonate, corresponding to the retention time of 3.43 min; D, detection of the enzymatic product of xylose dehydrogenase; E, detection of the enzymatic product of xylonolactonase; F, ion chromatogram of the extracellular metabolites of strain BL21ΔxylAB/pA-xdhxylC after being induced for 12 h. Both the enzymatic reaction mixtures and culture broth supernatant were appropriately diluted for ion chromatography analysis.

In several previous studies, a general high performance liquid chromatography (HPLC) method equipped with an ion exchange column (Aminex HPX-87H, Bio-Rad) was used to determine the extracellular metabolites including xylose, xylonate and xylonolactone. However, these three chemicals displayed similar retention times under the corresponding separation conditions, leading to the inaccuracy of measurement. Here, we developed an ion chromatography (IC) method to separate the three metabolites. Under such a condition, they were generally well separated, except that xylose was eluted only 0.14 min after xylonolactone (Figure 3A–C). Then the established IC analysis method was applied to determine the metabolites concentrations in the fermentation culture and the enzymatic products of xylose dehydrogenase and xylonolactonase. As the original xylose dehydrogenase activity has been measured by determining the absorption of NADH at 340 nm [16], we briefly detected the enzymatic reaction products of the enzyme by the established IC method. As shown in Figure 3D, the reaction mixture of xylose dehydrogenase gave an apparent peak of xylonolactone when xylose was supplemented as the substrate. And the reaction mixture of xylonolactonase showed the peaks of xylonolactone and xylonate, respectively (Figure 3E). These results demonstrated that both of the enzymes were expressed in their active forms in the recombinant *E. coli* strains.

### Comparison of Xylonate Productivities of Different Engineered Strains

**Figure 4:**
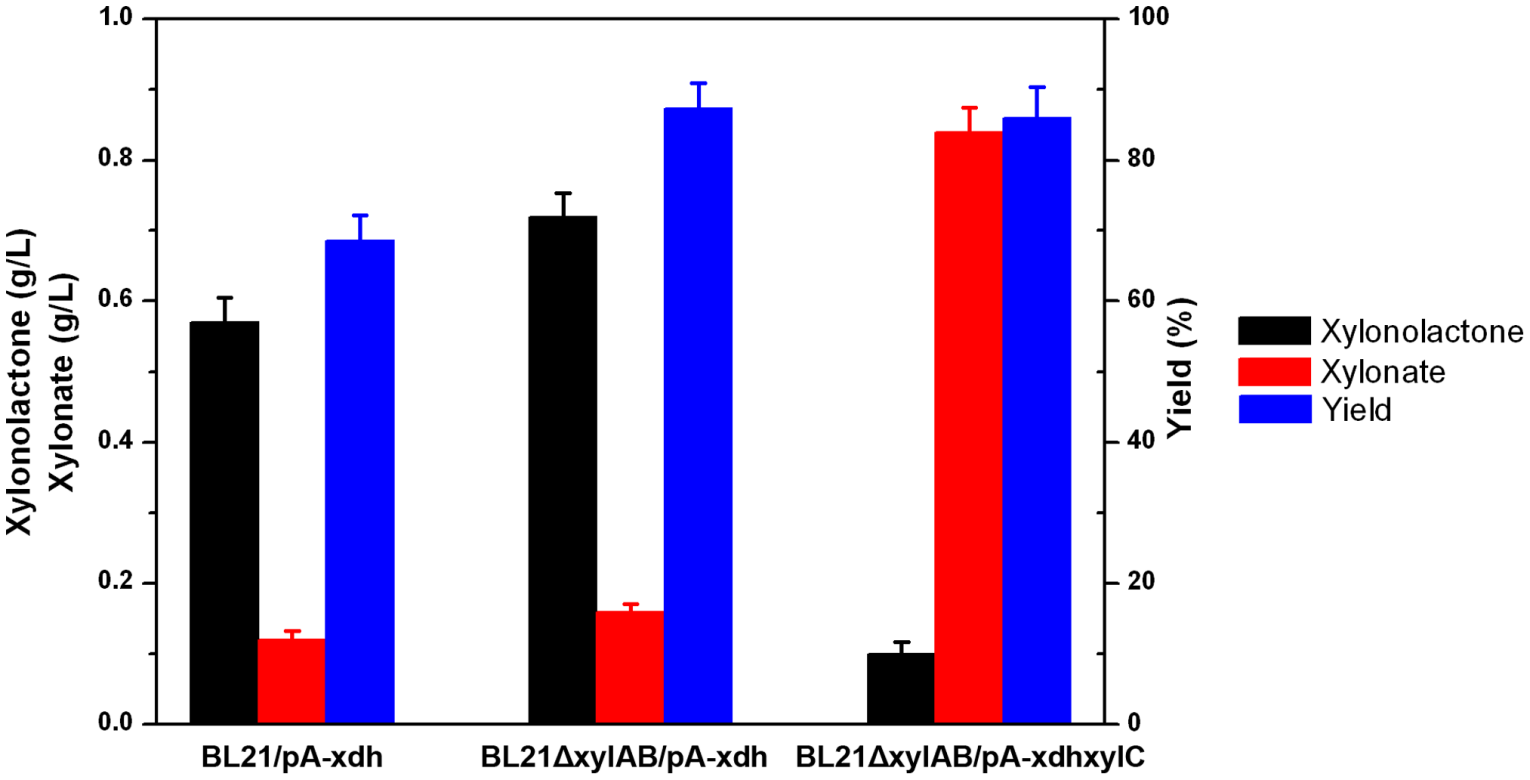
Comparison of xylonate and xylonolactone production of several different strains. Data were obtained after each strain was induced for 12 h in liquid LB medium supplemented with 1 g/L xylose. BL21/pA-xdh, strain BL21 star(DE3) expressing *C. crescentus* xylose dehydrogenase; BL21ΔxylAB/pA-xdh, knockout of native *xylA* and *xylB* while expressing *C. crescentus* xylose dehydrogenase; BL21ΔxylAB/pA-xdhxylC, knockout of native *xylA* and *xylB* while coexpressing *C. crescentus* xylose dehydrogenase and xylonolactonase.

Because the native xylonate-producing strains are not suitable for an industrial process, several heterologous systems have been developed for xylonate production, including *Saccharomyces cerevisiae* [22] and *Kluyveromyces lactis* [14]. Compared with these yeast strains, *E. coli* genome encodes several xylose transporters [23] and xylonate transport across *E. coli* cell membrane is also efficient, which might facilitate the consumption of xylose and the accumulation of xylonate. However, *E. coli* lacks the requisite xylose dehydrogenase activity, and thus wild-type *E. coli* strain could not convert xylose to xylonate. To construct a xylonate-producing strain, the xylose dehydrogenase from *C. crescentus* was heterologously expressed in *E. coli* BL21 star(DE3). As expected, accumulations of xylonolactone and xylonate were found in the cultures of the recombinant strain. In order to reduce the inhibiting effects of xylonolactone and facilitate the IC analysis of metabolites, the substrate xylose was supplemented at a relatively low concentration (1 g/L). As shown in Figure 4, 0.57 g/L xylonolactone and 0.12 g/L xylonate were produced after being induced for 12 h and the initial xylose was nearly completely exhausted. This productivity was much lower than the theoretical value because *E. coli* could catabolize xylose through the native phosphate pentose pathway [24]. To reduce the conversion of xylose to biomass, we further disrupted native *E. coli xylA* and *xylB* genes, which encode the first two enzymes responsible for xylose utilization [25]. When the native xylose catabolic pathway was blocked in the strain BL21ΔxylAB, the final titers of xylonolactone and xylonate were enhanced to 0.72 g/L and 0.16 g/L, respectively. However, most of the fermentation products were xylonolactone in the strain expressing the xylose dehydrogenase solely. In order to increase the hydrolysis rate of xylonolactone, we further constructed the engineered strain BL21ΔxylAB/pA-xdhxylC. The ion chromatograph of extracellular metabolites of this strain after 12 h-induction was shown in Figure 3F. We could find most of the xylonolactone was converted to xylonate by this strain. The final titers of xylonolactone and xylonate reached 0.10 g/L and 0.84 g/L and the molar yield of xylonolactone and xylonate on xylose also reached 86.0%.

### Xylonate Production Under Fed-batch Cultivation

**Figure 5:**
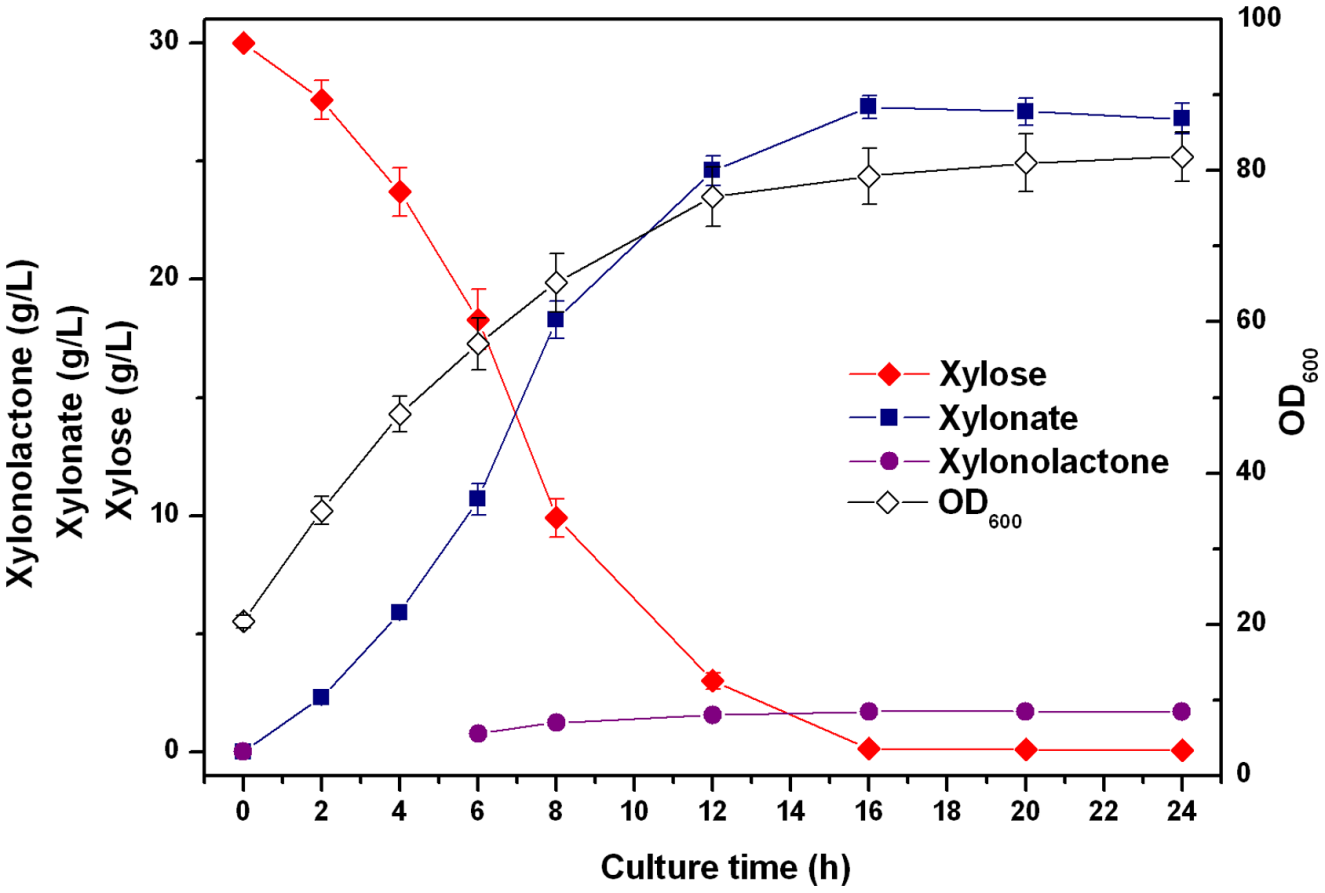
Time profiles for cell density (OD_600_), residual xylose, xylonate and xylonolactone concentrations in the culture broth during fed-batch culture of the finally engineered strain BL21ΔxylAB/pA-xdhxylC.

Among the industrially important microorganisms, *E. coli* has been used for various biotechnological processes and production of many valuable chemicals [26]. To investigate the feasibility for larger-scale production of xylonate, the finally engineered *E. coli* strain BL21ΔxylAB/pA-xdhxylC was cultured in a 5 L-scale laboratory fermenter. Cell density, xylose utilization, and products accumulation were monitored over the course of the experiment. Figure 5 shows the time profiles for cell density, residual xylose, xylonolactone and xylonate concentrations during the fermentation processes. For approximately 16 h post-induction, the bacteria grew very fast. Xylonate accumulated rapidly in the culture media while xylonolactone remained at a relatively low level. The highest xylonate production was obtained after 16 h induction, that is, 27.3 g/L. At this time, the final titer of xylonolactone reached 1.7 g/L and the initial xylose was completely exhausted. The volumetric productivity of xylonate and xylonolactone was 1.8 g/(L·h). The molar yield of xylonate and xylonolactone on xylose was about 88.0%, which was comparable to the shake-flask scale. Both of the titers of xylonate and xylonolactone remained stable during the following culture processes. In contrast, the recombinant strain only expressing xylose dehydrogenase (BL21ΔxylAB/pA-xdh) grew much slower after being induced with IPTG and cell growth ceased at an OD_600_ of around 40 even though glycerol was still being consumed (data not shown).

In two previous studies using yeast as the host organism, *S. cerevisiae* could only accumulate up to 3.8 g/L xylonate [13] while *K. lactis* produced 19 g/L xylonate from 40 g/L xylose [14]. Considering that yeast does not have an efficient pentose transport system, xylose uptake and metabolism might be limited in these strains. As for the native xylonate producers, the highest production was reported to be 92 g/L achieved by *P. fragi* [7], which was much higher than the *E. coli* strains and the yield of xylonate on xylose reached 92%, which was also comparable to the current study. This might be due to that the wild-type strains could endure high concentrations of xylonate. Therefore, the current strains have the potential to be further engineered to enhance their resistance to xylonate, thus leading to even higher production. On the other hand, *E. coli* grows much faster in cheap culture medium than the native producers [27]. The whole fermentation process only requires less than 24 h for the engineered strain in this work, while the native producing strains always take several days to reach the maximum titer. This would contribute to reducing the production cost during large-scale fermentation.

### Conclusions

In this study, a robust xylonate-producing *E. coli* strain was successfully constructed. Three distinct genetic alterations targeted at the xylose or xylonate metabolic pathways were introduced into the host strain BL21 star(DE3), including knockout of the endogenous *xylA* and *xylB* genes, which encodes the xylose isomerase and xylulose kinase, to block the native xylose catabolism; heterologous expression of a xylose dehydrogenase to render *E. coli* capable of converting xylose to xylonolactone; and further introducing a xylonolactonase to hydrolyze the intermediate xylonolactone to xylonate. Under fed-batch conditions, up to 27.3 g/L xylonate and 1.7 g/L xylonolactone were produced out of 30 g/L xylose by the finally engineered strain BL21ΔxylAB/pA-xdhxylC. The volumetric productivity was as high as 1.8 g/(L·h). This engineered *E. coli* has a promising application for the industrial-scale production of xylonate.

## Supporting Information

Figure S1 **Identification of the** ***xylA*** **and** ***xylB*** **knockout** ***E. coli*** **strains.** PCR verifications were performed with primers xylAB_DelIden_F and xylAB_DelIden_R (Table S1) corresponding to sequences up- and downstream of disrupted regions. Lane M, DNA molecular weight markers; lane 1, the original strain BL21 star(DE3); lane 2, the strain after introducing the kanamycin resistant disrupting cassette; lane 3, the final strain BL21/ΔxylAB by eliminating the kanamycin resistance by plasmid pCP20.
(TIF)

Table S1 **Primers used in this study for gene disruption, verification and plasmids construction.**
(DOC)
