# Supplementary figures and images for: Metabolic Engineering of Escherichia coli for the Production of Xylonate
Source: PLoS One. 2013 Jul 5;8(7):e67305. doi: 10.1371/journal.pone.0067305 (PMC3702539; doi:10.1371/journal.pone.0067305)

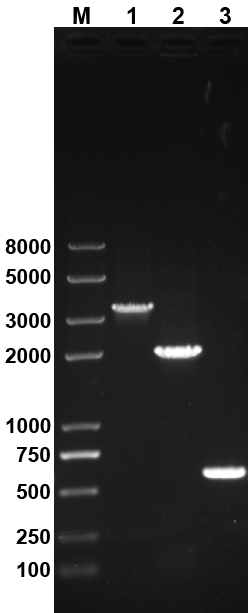

Supplement: Figure S1 — Identification of the xylA and xylB knockout E. coli strains. PCR verifications were performed with primers xylAB_DelIden_F and xylAB_DelIden_R (Table S1) corresponding to sequences up- and downstream of disrupted regions. Lane M, DNA molecular weight markers; lane 1, the original strain BL21 star(DE3); lane 2, the strain after introducing the kanamycin resistant disrupting cassette; lane 3, the final strain BL21/ΔxylAB by eliminating the kanamycin resistance by plasmid pCP20. (TIF) [file pone.0067305.s001.tif]
