# Supplementary material for: Metabolic Engineering of Escherichia coli for the Production of Xylonate
Source: PLoS One. 2013 Jul 5;8(7):e67305. doi: 10.1371/journal.pone.0067305 (PMC3702539; doi:10.1371/journal.pone.0067305)
Supplement: Table S1 — Primers used in this study for gene disruption, verification and plasmids construction. (DOC) [file pone.0067305.s002.doc]

| Oligonucleotide primers | Sequences |
| --- | --- |
| xylAB_Del_F | ATGCAAGCCTATTTTGACCAGCTCGATCGCGTTCGTTATgtgtaggctggagctgcttc |
| xylAB_Del_R | TTACGCCATTAATGGCAGAAGTTGCTGATAGAGGCGACGatgggaattagccatggtcc |
| xylAB_DelIden_F | CATGAGATCCATAGCCCAACC |
| xylAB_DelIden_R | TACCCAGTTTCATCATTCCATT |
| xdh_F_NcoI | CATGCCATGGGCATGTCCTCAGCCATCTATCCCAG |
| xdh_R_BamHI | CGCGGATCCTCAACGCCAGCCGGCGTCGATC |
| xylC_F_NdeI | GGGAATTCCATATGACCGCTCAGGTTACATGCG |
| XylC_R_XhoI | CCGCTCGAGTTAAACCAGACGAACTTCGTGC |
